# Cutaneous polyarteritis nodosa and pulmonary arterial hypertension: An unexpected liaison. A case report

**DOI:** 10.1097/MD.0000000000036563

**Published:** 2023-12-15

**Authors:** Elsa Berardi, Gianfranco Antonica, Annagrazia Procaccio, Donatello Marziliano, Nicola Susca, Patrizia Leone, Carlo Sabbà, Vito Racanelli, Marcella Prete

**Affiliations:** a Department of Interdisciplinary Medicine, Internal Medicine Unit, “Aldo Moro” University of Bari Medical School, Bari, Italy; b Centre for Medical Sciences, University of Trento and Internal Medicine Unit, Santa Chiara Hospital, Provincial Health Care Agency (APSS), Trento, Italy.

**Keywords:** case report, cPAN, necrotizing vasculitis, pulmonary arterial hypertension, skin vasculitis

## Abstract

**Background:**

Cutaneous polyarteritis nodosa (cPAN) is a form of medium-sized vessel necrotizing vasculitis. It is a rare, skin-limited variant of polyarteritis nodosa, characterized by dermal and subcutaneous tissue involvement. The most common findings in cPAN include digital gangrene, livedo reticularis, and tender subcutaneous nodules. However, while limited to the skin, cPAN results in significant morbidity and mortality due to the accompanying skin ischemia and necrosis, such that patients are vulnerable to superinfection. Here, we describe a unique presentation of cPAN associated with pulmonary arterial hypertension (PAH).

**Methods:**

A 78-year-old female presented with digital ischemia and leg ulcers associated with PAH. Skin biopsy showed necrotizing fibrinoid necrosis of the small- and middle-sized vessels of the dermis. A diagnosis of cPAN and PAH was made. The patient was treated with glucocorticoids, vasodilators, and cyclophosphamide.

**Results:**

She died due to severe sepsis complications.

**Conclusion:**

To date, this is the first case report describing the association between cPAN and PAH. In this case, PAH is a complication of the cutaneous vasculitides suggesting that vasculopathy could play a role in the pathophysiology of PAH. However, the underlying pathophysiological mechanisms still have to be firmly established.

## 1. Introduction

Cutaneous polyarteritis nodosa (cPAN) is a rare form of skin-limited necrotizing vasculitis that commonly affects the medium-sized arteries of the dermis and the subcutaneous tissue.^[1]^ Because cPAN does not include visceral involvement (i.e., kidneys, joints, muscles, nerves, and gastrointestinal tract), it is considered as a subset of PAN. While systemic PAN is more common in men, cPAN predominantly affects women.^[2]^ Despite its currently unknown etiology, the disease is likely mediated by an abnormal immune response triggered by infectious stimuli. It is often hepatitis B virus (HBV) related,^[3]^ whereas it has rarely been reported in association with hepatitis C virus (HCV) infection.^[4]^ cPAN has also been related to streptococcal infection, especially in children.^[5,6]^ Skin findings in cPAN are indistinguishable from those in PAN, although the main skin lesions in systemic PAN are purpura, livedo, and nodules, with recurrent reports of livedo reticularis or livedo racemosa and palpable subcutaneous nodules with ulcerations as well.^[7]^ Based on their different clinical courses, cPAN must be differentiated from PAN. However, as there are rare reports in which cPAN progressed to systemic PAN, all patients with cPAN should be closely monitored for possible complications and to rule out disease progression.

cPAN is commonly characterized by tender skin nodules, digital infarcts, and ulcers, although mild systemic symptoms and peripheral neuropathy limited to the area of skin involvement may overlap.^[8]^ Here we present the case of a patient with cPAN admitted for severe leg ulcers and finger ischemia. Asymptomatic pulmonary arterial hypertension (PAH) was diagnosed as well and the clinical evolution was atypical.

## 2. Case presentation

**Figure 1.**
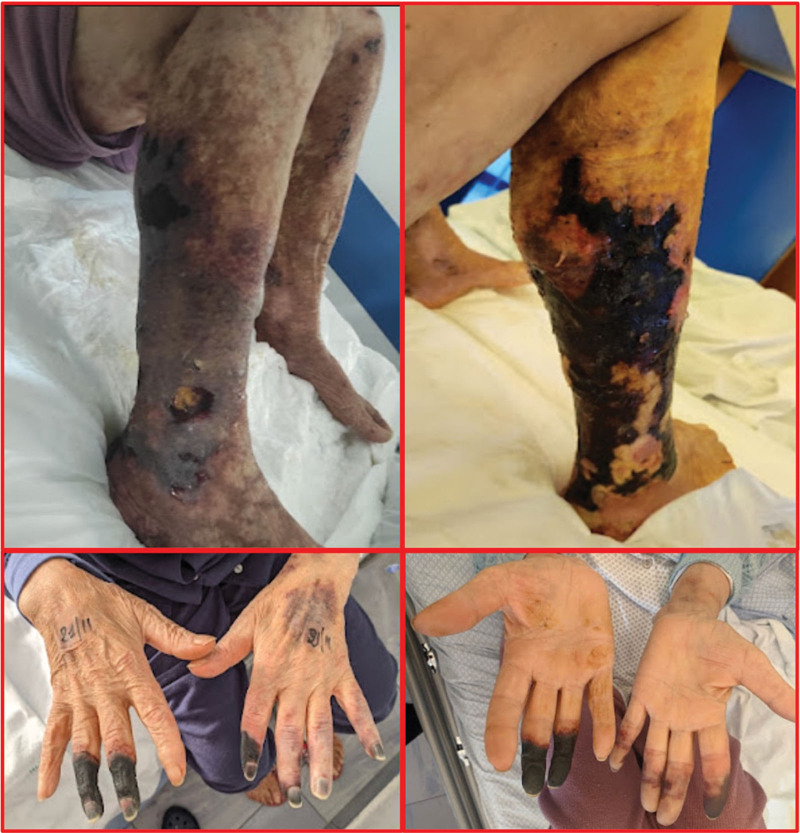
Extensive necrotic phenomena of upper and lower limbs, with leg ulcers and skin necrosis and fingers gangrene.

A 78-year-old female presented with right-hand 3rd to 4th finger ischemia and was admitted to the Internal Medicine Unit, Policlinic Hospital of Bari, in November 2022. Physical and dermatological examination revealed a bilateral erythematous rash on her cheeks, 3rd and 4th digital ischemia limited to the distal phalanx, and livedo reticularis of the lower limbs. Acral branched ulcerative lesions and coalescing at the lower limbs were observed bilaterally. The affected area showed diffuse purpuric perilesional skin with inflamed irregular margins associated with scar tissue (Fig. 1). The lesions were accompanied by severe pain and subjective tingling, together with edema of the lower limbs. The patient had a history of skin dyschromia and slight skin ulcers on the legs since 2020.

Her laboratory data were as follows: erythrocyte sedimentation rate 35 mm/h (normal value, 1–20 mm), C-reactive protein (18.2 mg/L), mild anemia (hemoglobin = 11.4 g/dL), and normal white blood cells and platelet count. Serum electrophoresis showed a nonquantifiable monoclonal component consisting of immunoglobulin G (IgG) κ versus polyclonal IgG, indicative of mixed cryoglobulinemia type 2, a 4% cryocrit, and hypogammaglobulinemia (8.8%). Her autoimmune serology results were negative for antineutrophil cytoplasmic antibody (ANCA) and antinuclear antibodies (ANA), as were the results of tests for neoplastic markers, including carcino-embrionic antigen, alpha fetoprotein, cancer antigen (CA), CA15.3, CA125, and CA19.9, and Cyfra 21.1. Lymphocyte immunophenotyping showed an increased CD19-CD20; the levels of all other tested markers were normal. However, she was positive for cryoagglutinins but negative for rheumatoid factor (RF), Bence-Jones protein, free light chains, and lupus anticoagulant. Her C3-C4 levels were normal. A 24-hour urine collection showed 280 mg per 24 hours and thus proteinuria. The results of blood and urine cultures, a severe acute respiratory syndrome coronavirus-2 swab and QuantiFERON test, and serology for legionella, mycoplasma, and chlamydia were all negative, but IgG positivity for toxoplasmosis, rubella, cytomegalovirus, herpes simplex virus 1-2, Varicella-Zoster virus, measles, Epstein-Barr virus, parvo B19, parotitis, adenovirus, and enterovirus were detected. Viral hepatitis testing by anti-HCV and HCV-RNA assays were negative, but serology for previous HBV infection was positive (positive anti-hepatitis B surface antigen antibody and anti-hepatitis B core antigen antibody, negative HBV DNA). An ulcer skin swab was positive for *Acinetobacter baumannii and Streptococcus agalactiae*.

A cardiac evaluation was performed as the patient complained of palpitations and exertional dyspnea. Multiple electrocardiogram tracings showed supraventricular extrasystoles, confirmed by Holter electrocardiogram. Ultrasonography (US) of the heart revealed a right convex-oriented atrial septal aneurysm without atrial septal defect and findings suggestive of PAH (systolic pulmonary arterial pressure = 60 + 5 mm Hg) with dilation (37 mm) of the main pulmonary artery. Pulmonary embolism was excluded given the absence of clinical symptoms and because the patient was already receiving anticoagulant treatment for previous deep-vein thrombosis of the lower limbs. Electromyography demonstrated limited sensorial-motor axonal neuropathy in the lower limbs.

**Figure 2.**
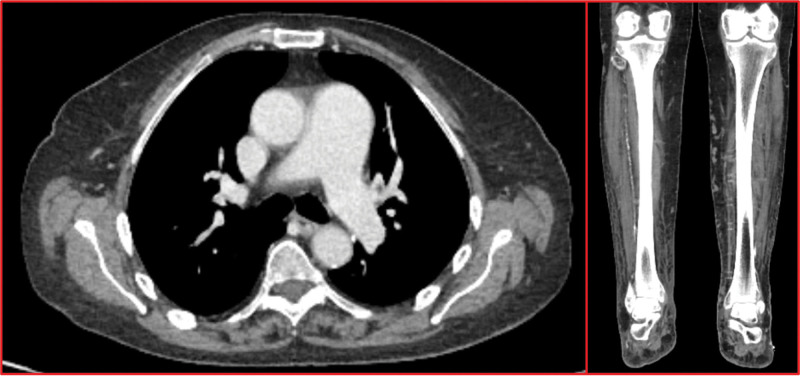
Contrast enhanced CT scan shows main pulmonary trunk enlargement, suggestive for pulmonary hypertension, and right anterior tibial artery narrowing with a beading appearance. CT = computed tomography.

Full body computed tomography ruled out other secondary causes of PAH (such as left heart disease, chronic thromboembolic pulmonary hypertension, and portopulmonary hypertension) and lung parenchymal involvement and instead exclusively showed right anterior tibial artery beading, with a “rosary crown” appearance, suggestive of mid-sized vessel vasculitis (Fig. 2). However, these findings were not confirmed by the US-Doppler examination of the aorta, its main branches, and the upper and lower limb arteries. Atherosclerotic plaques were observed but they were not hemodynamically significant. The right anterior tibial artery was of irregular caliber but without critical stenosis, occlusion, or aneurysms.

**Figure 3.**
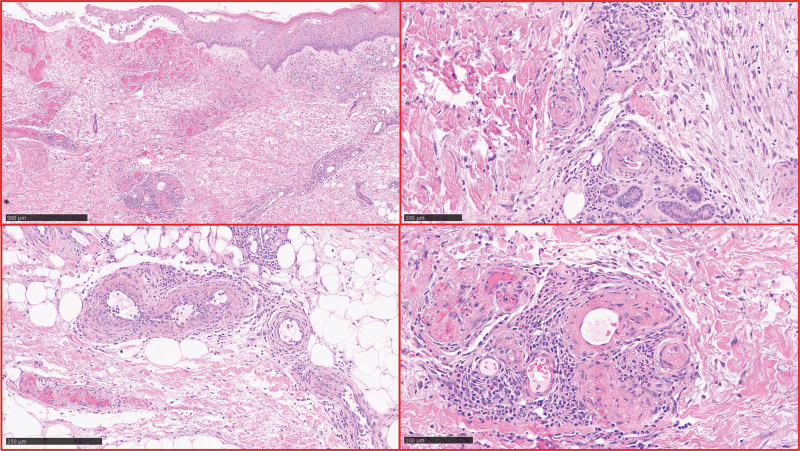
Microscopic skin biopsy sample, stained with hematoxylin/eosin. The figures at different magnifications show typical histopathological cutaneous features of PAN: necrotic ulcer, dermal edema, and swelling of the small vessel wall associated with quite evident fibrinoid necrosis, mild perivasal infiltration of neutrophils, and signs of endothelial damage. PAN = polyarteritis nodosa.

At 2 weeks from admission, the patient developed ischemia of the index and middle fingers of the left hand. Capillaroscopy showed neoangiogenic areas but no megacapillaries or microhemorrhages. These findings were compatible with ischemic microangiopathy and raised a clinical suspicion of vasculitis. Systemic lupus erythematosus-related and ANCA-associated vasculitis were unlikely, due to the absence of respiratory involvement and the patient’s ANA and ANCA negativity. Cryoglobulinemic vasculitis was considered, based on the detection of type II mixed cryoglobulinemia found at serum capillary electrophoresis, but it was ruled out due to normal C3 and C4 levels, negative HCV serology, and RF negativity. Incisional skin biopsies of the right calf (0.7 cm × 0.3 cm × 0.3 cm) and right malleolar (1.4 × 1 × 0.5) lesions were performed. The histological examination showed ulcerated fibrinoid necrosis with granulocyte exocytosis of small- and mid-sized vessels associated with vessel wall fibrinoid necrosis, perivascular lymphocyte and granulocyte infiltration, diffuse hemorrhagic dermal infiltration, and negative periodic acid-Schiff staining (Fig. 3). These findings were compatible with a clinical diagnosis of cPAN, since the patient presented with pure cutaneous manifestations without systemic involvement.

Secondary causes of vasculitis or systemic involvement were excluded in a positron emission tomography-computed tomography scan, which revealed left temporo-preauricular area hypercaptation (maximum standardized uptake value = 31.9). The area was further evaluated with US-Doppler, which identified a hypoechogenic nonvascularized lymph node with a wide cortical area and hilum as well as a coalescing round lymph node with thickened walls (maximum diameter 6 mm). A US-guided biopsy was subsequently performed. The histological report described a well-vascularized area associated with proximal mesenchymal cell proliferation without atypia. However, given the insufficiency of the sample, it was not possible to establish a diagnosis.

The patient was started on methylprednisolone (60 mg), with cyclophosphamide (50 mg/d) then added. Due to the severity of the skin ulcers, she also received a prostacyclin (prostaglandin I2) analog (iloprost 0.05 mg in 500 cc NaCl 0.9% at 40 mL/h administered in 12 h for 4 wk). Treatment was effective in demarcating the necrosis and provided better control of the pulmonary hypertension but it did not significantly improve the ulcers, given their extent and advanced stage. She was discharged in stable clinical condition and referred to our outpatient clinic for follow-up before her readmission for cardiac catheterization and fingertip amputation. However, the patient was readmitted following an episode of high-frequency (170 bpm) atrial fibrillation during an outpatient evaluation. During hospitalization, because of her frailty and the immunosuppressive treatment, she developed multidrug-resistant *Acinetobacter* sepsis and died.

This case was reported to provide critical information for an unusual clinical feature and share our experience, for optimizing management and treatment of cPAN patients. Our clinical observation might lead to further trials and the identification of new pathogenetic mechanisms associated with cPAN and PAH.

## 3. Discussion

The precise etiology of cPAN is unknown, although it is thought to be immune-complex mediated. Several infectious and noninfectious conditions are associated with the initiation and relapse of the disease. Streptococcal infection has also been implicated, but other infectious agents, such as parvovirus B19, mycobacterium, and hepatitis B and C viruses, may also be involved. Occasionally, a recessive loss-of-function mutation in the *CECR1* gene, encoding deaminase A, has been associated with cPAN in children, pointing to a genetic susceptibility.^[9,10]^ Whereas PAN patients may present with findings indicating visceral involvement,^[1]^ in cPAN, the lesions are limited to the skin, adjacent muscles, nerves, and joints. Patients with cPAN typically present with tender subcutaneous nodules, livedo reticularis, and ulceration, mostly localized to the lower extremities. The tender, erythematous subcutaneous nodules, with a diameter of 0.5 to 3 cm, usually either ulcerate or disappear spontaneously. Other findings include petechiae, purpura, cutaneous necrosis, autoamputations, and local extracutaneous manifestations, such as arthralgia, myalgia, constitutional symptoms (e.g., fever and malaise), and peripheral neuropathy (mononeuropathy and mononeuritis multiplex). Patients may complain of neurologic symptoms. The most common neurologic deficit in patients with PAN is mononeuritis multiplex, which occurs in nearly 70% and typically involves the radial, ulnar, and peroneal nerves. Additionally, patients may experience sensory and motor impairments. As there are no specific clinical findings, the diagnosis is based on clinical features of isolated skin involvement, confirmed by histopathology. A deep incisional biopsy, including subcutaneous tissue, is necessary for an accurate diagnosis of the disease. The pathology report will highlight leukocytoclastic vasculitis in the small- to medium-sized arterioles of the deep dermis or hypodermis, with or without associated fibrinoid necrosis.^[11]^ Nevertheless, necrotizing arteritis of the small- and medium-sized vessels is a histopathological feature that occurs in both cPAN and systemic PAN. Autoantibody testing is not likely to aid in confirming the diagnosis of cPAN,^[12]^ although negative results help to exclude other types of systemic vasculitis. There is also no specific serologic test that confirms a diagnosis of cPAN but laboratory tests can support assessments of end-organ damage, therefore excluding systemic involvement in patients with clinical and skin biopsy findings suggestive of PAN. A basic laboratory work-up encompasses a complete blood count, basic metabolic panel, liver function tests, and urinalysis with microscopic evaluation. Additional tests are recommended to rule out other types of vasculitis affecting medium-sized vessels. The following studies are indicated for all patients: ANA, ANCA, RF, serum cryoglobulins, and complement components (C3 and C4). While these tests are typically negative in PAN, they enable the establishment of a differential diagnosis that includes ANCA-associated vasculitis, cryoglobulinemic vasculitis (RF, cryoglobulins, and complement levels), and rheumatoid vasculitis (RF, anti-cyclic citrullinated peptide antibodies).

In a retrospective analysis of 31 cPAN cases collected in a Spanish dermatological center, only two-thirds of the patients had at least one extracutaneous systemic manifestation and none had PAH as a comorbidity.^[13]^ Ulceration together with an elevation of inflammatory parameters predicted a worse prognosis.^[13]^ In our patient, the finding of PAH was incidental at the clinical onset of cPAN. In the diagnostic work-up, other common causes of PAH were ruled out and an underlying vascular process remained the only possible cause or casual association.^[14]^

PAH is a well-known complication of connective tissue diseases, such as systemic sclerosis, systemic lupus erythematosus, mixed connective tissue disease, and, albeit rarely, rheumatoid arthritis, dermatopolymyositis, and Sjogren syndrome, in which it complicates the clinical course and represents a prognostic constraint, particularly in systemic sclerosis.^[15]^ In a literature search, we found only a single case report of PAH secondary to ANCA-associated vasculitis, which developed in a young woman.^[16]^

PAH has not been previously related to cPAN or systemic PAN. Bronchial artery aneurysms, pleural effusion, diffuse alveolar damage, and hemorrhage are pulmonary conditions and/or complications that have been rarely described in PAN.^[17]^ Thus, PAH in our patient remained an unexplained finding, since she did not live long enough to undergo cardiac catheterization.^[18]^

Given the milder symptoms in cPAN, treatment usually consists of colchicine (0.6 mg twice daily) or dapsone (50–150 mg daily), administered for several weeks.^[19]^ Corticosteroids are the cornerstone of treatment for cPAN and are used in many patients with severe disease.^[19]^ The optimal duration of treatment is uncertain. Immunosuppressive agents, such as cyclophosphamide, azathioprine, and methotrexate, can be used in patients unresponsive to steroid therapy. As cPAN often has a chronic-relapsing and remitting course, initiating immunosuppressant at the same time as steroid therapy may be useful and enable a lower overall lifetime exposure to steroids.^[20]^

In patients with resistant or relapsed disease, the role of intravenous immunoglobulin as a successful option for the treatment of skin lesions has been highlighted in 2 case reports.^[21,22]^ Moreover, the effectiveness of vasodilators and antithrombotic agents, such as prostaglandin I2 analogues, has been reported, especially in the treatment of the skin ulcers and gangrene associated with cPAN^[23]^ and other types of vasculitis.^[24]^

Less aggressive treatments may be warranted in cPAN, as it is not usually associated with life-threatening or progressive outcomes. However, a poorer prognosis in cPAN may be associated with factors such as pretreatment ulcer status, neutrophil to lymphocyte ratio, C-reactive protein level, and the immune systemic inflammation index^[25]^ or comorbidities, such as PAH.^[26]^

While the systemic symptoms in cPAN, such as arthralgias, fever, and malaise, are generally mild, complications, including ulceration, digital infarction, and even autoamputation, can significantly affect the quality of life. The necrotic tissue may be an entry site for bacterial infection, which places the patient at high risk of infection, made worse by concomitant immunosuppressive treatment, and thus at significant risk of morbidity and perhaps even death. Unlike the majority of cases of cPAN in the literature, in which the course was benign but recurring, our patient developed a progressive worsening of her disease until she finally died from it.

The present report carries some limitations. How much the concomitant presence of PAH affected the outcome remains unclear. Moreover, we were not able to perform a thorough evaluation of the PAH of the patient (e.g., cardiac catheterization) because her clinical condition rapidly worsened leading to death. In this regard, we could not properly assess the therapy efficacy, because she died before immune suppressant treatment could show its effects.

In conclusion, for patients with cutaneous necrotizing vasculitis, especially when the etiology is not clear, an underlying condition of PAH condition should be ruled out. Echocardiographic evaluation is required as part of the assessment. Some PAH are probably due, at least in part, to direct pulmonary artery involvement by the vasculitic process.

## 4. Conclusions

cPAN is a rare form of cutaneous vasculitis of unknown etiology with a chronic-relapsing course and features that differ from those of systemic PAN. In our patient, the aim was to treat cPAN with immune suppressive treatment at baseline and with vasodilators to resolve the ischemia. The latter approach, unfortunately, did not improve the patient’s skin lesions but only arrested their progression and helped to control her PAH. As this is the first reported case of PAH associated with cPAN, it demonstrates the importance of a close follow-up of cPAN patients, as multiple complications may arise, such as PAH. Larger randomized prospective trials are needed to further evaluate the possible pathogenetic mechanism of this association.

## Author contributions

**Data curation:** Elsa Berardi, Gianfranco Antonica, Annagrazia Procaccio.

**Investigation:** Elsa Berardi, Gianfranco Antonica, Annagrazia Procaccio, Donatello Marziliano.

**Writing – original draft:** Elsa Berardi, Annagrazia Procaccio, Donatello Marziliano.

**Writing – review & editing:** Gianfranco Antonica, Nicola Susca, Patrizia Leone, Carlo Sabbà, Vito Racanelli, Marcella Prete.

**Conceptualization:** Nicola Susca, Patrizia Leone, Vito Racanelli, Marcella Prete.

**Formal analysis:** Carlo Sabbà, Vito Racanelli, Marcella Prete.

**Supervision:** Carlo Sabbà, Vito Racanelli, Marcella Prete.

**Methodology:** Vito Racanelli, Marcella Prete.
